# 1,5-Diphenyl­carbonohydrazide *N*,*N*-di­methyl­formamide

**DOI:** 10.1107/S160053681003922X

**Published:** 2010-10-09

**Authors:** Ai-yun Zhang, Si-yao Ma, Dong Bu

**Affiliations:** a Department of Physics and Chemistry, Henan Polytechnic University, Jiaozuo 454000, People’s Republic of China; b Department of Mathematics, Xi’an University of Architecture and Technology, Xi’an 710055, People’s Republic of China

## Abstract

In the title compound, C_13_H_14_N_4_O·C_3_H_7_NO, a 1,5-phenyl­carbonohydrazide mol­ecule cocrystallizes with an *N*,*N*-dimethyl­formamide mol­ecule. In the 1,5-phenyl­carbonohydrazide mol­ecule, the two phenyl rings are twisted by an angle of 45.8 (5)°. Inter­molecular N—H⋯O hydrogen bonds and weak inter­molecular C—H⋯O inter­actions contribute to a supra­molecular two-dimensional network in the (101) plane.

## Related literature

For literature on the applications of 1,5-diphenyl­carbonohydrazide, an artificial electron-donor material, see: Verma & Singh (1995 ▶); Melis *et al.* (1992 ▶); Prasad *et al.* (1991 ▶); Sundari & Raghavendra (1990 ▶); Mishra *et al.* (1993 ▶). For the structure of diphenyl­carbonohydrazide, see: De Ranter *et al.* (1979 ▶). For related structures, see: Hamuro *et al.* (1999 ▶); Jian *et al.* (2003 ▶); Wei *et al.*(2006 ▶); Wang *et al.* (2001 ▶).
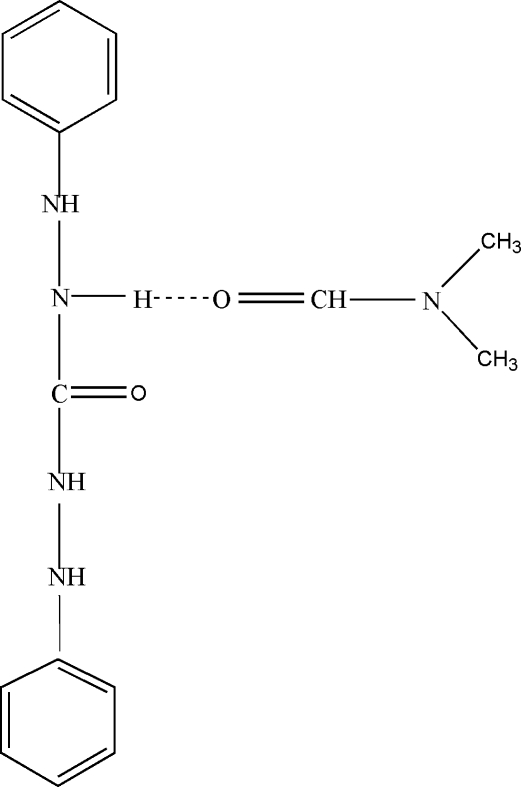


## Experimental

### 

#### Crystal data

C_13_H_14_N_4_O·C_3_H_7_NO
*M* *_r_* = 315.38
Monoclinic, 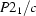

*a* = 5.9774 (2) Å
*b* = 14.8531 (6) Å
*c* = 18.4827 (7) Å
β = 96.029 (3)°
*V* = 1631.87 (11) Å^3^
*Z* = 4
Mo *K*α radiation
μ = 0.09 mm^−1^
*T* = 296 K
0.21 × 0.20 × 0.18 mm

#### Data collection

Bruker APEXII CCD area-detector diffractometer
Absorption correction: multi-scan (*SADABS*; Bruker, 2007 ▶) *T*_min_ = 0.982, *T*_max_ = 0.984
23310 measured reflections
2902 independent reflections
2061 reflections with *I* > 2σ(*I*)
*R*_int_ = 0.049

#### Refinement

*R*[*F* ^2^ > 2σ(*F* ^2^)] = 0.058
*wR*(*F* ^2^) = 0.193
*S* = 1.06
2902 reflections
209 parameters
H-atom parameters constrained
Δρ_max_ = 0.32 e Å^−3^
Δρ_min_ = −0.36 e Å^−3^

### 

Data collection: *APEX2* (Bruker, 2007 ▶); cell refinement: *APEX2*; data reduction: *SAINT* (Bruker, 2007 ▶); program(s) used to solve structure: *SHELXS97* (Sheldrick, 2008 ▶); program(s) used to refine structure: *SHELXL97* (Sheldrick, 2008 ▶); molecular graphics: *SHELXTL* (Sheldrick, 2008 ▶); software used to prepare material for publication: *SHELXTL*.

## Supplementary Material

Crystal structure: contains datablocks global, I. DOI: 10.1107/S160053681003922X/jj2045sup1.cif

Structure factors: contains datablocks I. DOI: 10.1107/S160053681003922X/jj2045Isup2.hkl

Additional supplementary materials:  crystallographic information; 3D view; checkCIF report

## Figures and Tables

**Table 1:** Hydrogen-bond geometry (Å, °)

*D*—H⋯*A*	*D*—H	H⋯*A*	*D*⋯*A*	*D*—H⋯*A*
N1—H1*A*⋯O1^i^	0.86	2.13	2.975 (3)	166
C6—H6*A*⋯O2^ii^	0.93	2.58	3.370 (4)	143
N2—H2*B*⋯O1^iii^	0.86	2.45	3.121 (3)	135
N3—H3*B*⋯O2	0.86	2.12	2.895 (3)	149
N4—H4*B*⋯O2^ii^	0.86	2.31	3.079 (3)	148

Symmetry codes: (i) 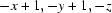; (ii) 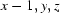; (iii) 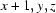.

**Fig. 1.**
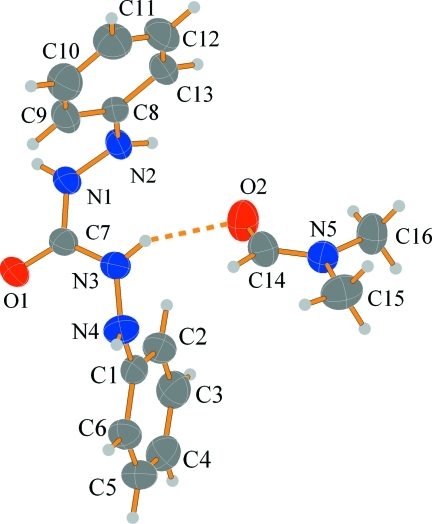
The molecular structure of the title compound, C16H21N5O2, showing the atom-labeling scheme with displacement ellipsoids drawn at the 50% probability level.

**Fig. 2.**
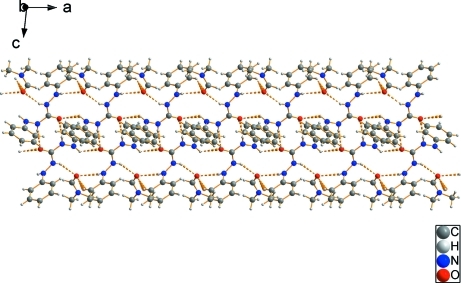
Packing diagram for the title compound viewed down the b axis. Dashed lines indicate N—H···O and C—H···O hydrogen bonds forming an infinite two-dimensional polymeric chain along the a axis.

## supplementary crystallographic information

### Comment

1, 5-diphenylcarbonohydrazide, an artificial electron-donor material, has a variety of applications (Verma & Singh, 1995; Melis *et al.*, 1992; Prasad *et al.*, 1991; Sundari & Raghavendra, 1990; Mishra *et al.*, 1993). The structure of diphenylcarbonohydrazide (C_13_H_14_N_4_O), (De Ranter *et al.*, 1979) and a number of diphenylcarbonohydrazide derivatives have been prepared (Jian *et al.*, 2003; Wang *et al.*, 2001; Hamuro *et al.*, 1999; Wei *et al.*, 2006).

The title compound, is a co-crystal with a 1,5-phenylcarbonohydrazide molecule and a *N*, *N*-dimethylformamide molecule in the unit cell (Fig. 1). In the 1,5-phenylcarbonohydrazide molecule, the dihedral angles of the two benzene rings are twisted by an angle of 45.8 (5)°.  The C7/N1/N2/C8 (-91.6 (3)°) and C7/N3/N4/C1 (75.3 (3)°) torsion angles confirm this twist. Crystal packing is dominated by N—H···O hydrogen bonds and weak C—H···O intermolecular interactions (Table 1) which contribute to a supermolecular 2-D network formed in the 101 plane (Fig. 2).

### Experimental

A mixture of 1, 5-diphenylcarbazide (0.0233 g), Cd(NO_3_)_2_(0.0132 g), *N*, *N*-dimethylformamide (5 ml), and water (12 ml) was stirred at room temperature for 6 h.  The solution was filtered and the filtrate was left to stand undisturbed. Upon slow evaporation at room temperature, the title compound appeared about a month later. The title compound was filtered, washed with water and dried at 298K. The single crystals were grown by slow evaporation of water and *N*, *N*-dimethylformamide in the filtered mixture of 1, 5-diphenylcarbazide, Cd(NO_3_)_2_, *N*, *N*-dimethylformamide, and water at 298K.

### Refinement

All of the H atoms were placed in their calculated positions and then refined using the riding model with Atom—H lengths of 0.93Å (CH), 0.96Å (CH_3_) or 0.86Å (NH).  Isotropic displacement parameters for these atoms were set to 1.2 times (NH), 1.2 (CH) or 1.2 (CH_3_) times *U*_eq_ of the parent atom.

### Crystal data

**Table:**

C_13_H_14_N_4_O·C_3_H_7_NO	*F*(000) = 672
*M**_r_* = 315.38	*D*_x_ = 1.284 Mg m^−^^3^
Monoclinic, *P*2_1_/*c*	Mo *K*α radiation, λ = 0.71073 Å
Hall symbol: -P 2ybc	Cell parameters from 3501 reflections
*a* = 5.9774 (2) Å	θ = 2.6–21.5°
*b* = 14.8531 (6) Å	µ = 0.09 mm^−^^1^
*c* = 18.4827 (7) Å	*T* = 296 K
β = 96.029 (3)°	Block, colorless
*V* = 1631.87 (11) Å^3^	0.21 × 0.20 × 0.18 mm
*Z* = 4	

### Data collection

**Table:**

Bruker APEXII CCD area-detector diffractometer	2902 independent reflections
Radiation source: fine-focus sealed tube	2061 reflections with *I* > 2σ(*I*)
graphite	*R*_int_ = 0.049
φ and ω scans	θ_max_ = 25.1°, θ_min_ = 1.8°
Absorption correction: multi-scan (*SADABS*; Bruker, 2007)	*h* = −7→7
*T*_min_ = 0.982, *T*_max_ = 0.984	*k* = −17→17
23310 measured reflections	*l* = −22→22

### Refinement

**Table:**

Refinement on *F*^2^	Secondary atom site location: difference Fourier map
Least-squares matrix: full	Hydrogen site location: inferred from neighbouring sites
*R*[*F*^2^ > 2σ(*F*^2^)] = 0.058	H-atom parameters constrained
*wR*(*F*^2^) = 0.193	*w* = 1/[σ^2^(*F*_o_^2^) + (0.1085*P*)^2^ + 0.5441*P*] where *P* = (*F*_o_^2^ + 2*F*_c_^2^)/3
*S* = 1.06	(Δ/σ)_max_ < 0.001
2902 reflections	Δρ_max_ = 0.32 e Å^−^^3^
209 parameters	Δρ_min_ = −0.36 e Å^−^^3^
0 restraints	Extinction correction: *SHELXL97* (Sheldrick, 2008), Fc^*^=kFc[1+0.001xFc^2^λ^3^/sin(2θ)]^-1/4^
Primary atom site location: structure-invariant direct methods	Extinction coefficient: 0.014 (4)

### Special details

**Table:**

Geometry. All esds (except the esd in the dihedral angle between two l.s. planes) are estimated using the full covariance matrix. The cell esds are taken into account individually in the estimation of esds in distances, angles and torsion angles; correlations between esds in cell parameters are only used when they are defined by crystal symmetry. An approximate (isotropic) treatment of cell esds is used for estimating esds involving l.s. planes.
Refinement. Refinement of *F*^2^ against ALL reflections. The weighted *R*-factor *wR* and goodness of fit *S* are based on *F*^2^, conventional *R*-factors *R* are based on *F*, with *F* set to zero for negative *F*^2^. The threshold expression of *F*^2^ > σ(*F*^2^) is used only for calculating *R*-factors(gt) *etc*. and is not relevant to the choice of reflections for refinement. *R*-factors based on *F*^2^ are statistically about twice as large as those based on *F*, and *R*- factors based on ALL data will be even larger.

### Fractional atomic coordinates and isotropic or equivalent isotropic displacement parameters (Å^2^)

**Table:**

	*x*	*y*	*z*	*U*_iso_*/*U*_eq_	
C1	0.4510 (4)	0.55844 (17)	0.25962 (14)	0.0456 (6)	
C2	0.6356 (5)	0.5015 (2)	0.26959 (15)	0.0573 (8)	
H2A	0.7563	0.5097	0.2423	0.069*	
C3	0.6407 (5)	0.4328 (2)	0.31971 (17)	0.0633 (8)	
H3A	0.7645	0.3946	0.3256	0.076*	
C4	0.4651 (5)	0.4197 (2)	0.36131 (16)	0.0639 (8)	
H4A	0.4689	0.3728	0.3947	0.077*	
C5	0.2840 (5)	0.4774 (2)	0.35257 (16)	0.0608 (8)	
H5A	0.1658	0.4698	0.3809	0.073*	
C6	0.2757 (4)	0.54590 (18)	0.30260 (15)	0.0509 (7)	
H6A	0.1521	0.5842	0.2974	0.061*	
C7	0.5425 (4)	0.57813 (16)	0.09720 (13)	0.0401 (6)	
C8	0.8908 (4)	0.71277 (16)	0.02790 (12)	0.0396 (6)	
C9	0.6983 (4)	0.74404 (19)	−0.01259 (15)	0.0518 (7)	
H9A	0.5677	0.7095	−0.0165	0.062*	
C10	0.7002 (5)	0.8262 (2)	−0.04697 (17)	0.0646 (8)	
H10A	0.5706	0.8467	−0.0742	0.078*	
C11	0.8908 (5)	0.8785 (2)	−0.04161 (17)	0.0665 (9)	
H11A	0.8907	0.9339	−0.0650	0.080*	
C12	1.0807 (5)	0.8478 (2)	−0.00140 (17)	0.0637 (8)	
H12A	1.2104	0.8828	0.0023	0.076*	
C13	1.0828 (4)	0.76617 (19)	0.03362 (15)	0.0506 (7)	
H13A	1.2129	0.7466	0.0612	0.061*	
C14	0.9098 (5)	0.78263 (19)	0.25852 (16)	0.0550 (7)	
H14A	0.7616	0.8000	0.2453	0.066*	
C15	0.9162 (6)	0.8990 (2)	0.34963 (18)	0.0675 (9)	
H15A	1.0221	0.9236	0.3871	0.101*	
H15B	0.7887	0.8755	0.3710	0.101*	
H15C	0.8675	0.9454	0.3154	0.101*	
C16	1.2467 (5)	0.8018 (2)	0.33949 (17)	0.0656 (8)	
H16A	1.3015	0.8413	0.3785	0.098*	
H16B	1.3423	0.8060	0.3010	0.098*	
H16C	1.2467	0.7410	0.3571	0.098*	
N1	0.7115 (3)	0.57302 (14)	0.05309 (11)	0.0445 (5)	
H1A	0.7038	0.5346	0.0181	0.053*	
N2	0.8964 (3)	0.63018 (13)	0.06480 (11)	0.0437 (5)	
H2B	1.0117	0.6151	0.0941	0.052*	
N3	0.5839 (4)	0.63150 (15)	0.15609 (11)	0.0510 (6)	
H3B	0.6994	0.6664	0.1597	0.061*	
N4	0.4411 (4)	0.63076 (15)	0.21120 (12)	0.0536 (6)	
H4B	0.3483	0.6743	0.2152	0.064*	
N5	1.0222 (3)	0.82726 (15)	0.31247 (11)	0.0483 (6)	
O1	0.3662 (3)	0.53634 (12)	0.08206 (10)	0.0491 (5)	
O2	0.9826 (3)	0.72042 (14)	0.22424 (12)	0.0664 (6)	

### Atomic displacement parameters (Å^2^)

**Table:**

	*U*^11^	*U*^22^	*U*^33^	*U*^12^	*U*^13^	*U*^23^
C1	0.0427 (14)	0.0537 (16)	0.0404 (14)	−0.0021 (11)	0.0043 (11)	−0.0108 (11)
C2	0.0430 (15)	0.073 (2)	0.0570 (17)	0.0041 (13)	0.0103 (13)	−0.0061 (15)
C3	0.0531 (17)	0.076 (2)	0.0592 (18)	0.0144 (14)	−0.0027 (14)	0.0009 (15)
C4	0.0681 (19)	0.0691 (19)	0.0538 (17)	−0.0009 (16)	0.0025 (15)	0.0077 (15)
C5	0.0565 (17)	0.072 (2)	0.0562 (17)	−0.0062 (15)	0.0179 (14)	−0.0011 (15)
C6	0.0445 (15)	0.0541 (16)	0.0558 (16)	0.0024 (12)	0.0129 (12)	−0.0057 (13)
C7	0.0391 (13)	0.0404 (13)	0.0411 (13)	−0.0030 (10)	0.0052 (10)	0.0033 (10)
C8	0.0367 (13)	0.0462 (14)	0.0367 (13)	−0.0037 (10)	0.0078 (10)	−0.0056 (10)
C9	0.0366 (14)	0.0610 (17)	0.0564 (16)	−0.0053 (12)	−0.0023 (12)	0.0045 (13)
C10	0.0560 (18)	0.071 (2)	0.0651 (19)	0.0068 (15)	−0.0035 (14)	0.0140 (15)
C11	0.073 (2)	0.0569 (18)	0.069 (2)	−0.0022 (15)	0.0072 (16)	0.0169 (15)
C12	0.0573 (18)	0.0631 (19)	0.071 (2)	−0.0181 (14)	0.0092 (15)	0.0067 (16)
C13	0.0392 (14)	0.0584 (17)	0.0536 (16)	−0.0074 (12)	0.0012 (12)	0.0020 (13)
C14	0.0434 (15)	0.0590 (17)	0.0609 (17)	0.0041 (13)	−0.0024 (13)	0.0002 (14)
C15	0.084 (2)	0.0539 (17)	0.0676 (19)	0.0083 (16)	0.0240 (17)	−0.0042 (15)
C16	0.0593 (18)	0.072 (2)	0.0623 (18)	0.0048 (15)	−0.0112 (15)	−0.0066 (15)
N1	0.0410 (11)	0.0466 (12)	0.0468 (12)	−0.0116 (9)	0.0092 (9)	−0.0087 (9)
N2	0.0345 (10)	0.0501 (13)	0.0453 (12)	−0.0064 (9)	−0.0018 (9)	0.0027 (9)
N3	0.0486 (13)	0.0596 (14)	0.0468 (12)	−0.0145 (10)	0.0146 (10)	−0.0109 (10)
N4	0.0550 (13)	0.0573 (14)	0.0512 (13)	0.0062 (11)	0.0181 (11)	−0.0043 (11)
N5	0.0454 (12)	0.0487 (13)	0.0503 (13)	0.0043 (10)	0.0024 (10)	−0.0043 (10)
O1	0.0416 (10)	0.0540 (11)	0.0520 (11)	−0.0122 (8)	0.0061 (8)	−0.0046 (8)
O2	0.0627 (13)	0.0650 (13)	0.0687 (13)	0.0034 (10)	−0.0060 (10)	−0.0200 (11)

### Geometric parameters (Å, °)

**Table:**

C1—C2	1.388 (4)	C11—C12	1.368 (4)
C1—C6	1.392 (3)	C11—H11A	0.9300
C1—N4	1.395 (3)	C12—C13	1.374 (4)
C2—C3	1.376 (4)	C12—H12A	0.9300
C2—H2A	0.9300	C13—H13A	0.9300
C3—C4	1.378 (4)	C14—O2	1.226 (3)
C3—H3A	0.9300	C14—N5	1.321 (4)
C4—C5	1.376 (4)	C14—H14A	0.9300
C4—H4A	0.9300	C15—N5	1.449 (3)
C5—C6	1.371 (4)	C15—H15A	0.9600
C5—H5A	0.9300	C15—H15B	0.9600
C6—H6A	0.9300	C15—H15C	0.9600
C7—O1	1.230 (3)	C16—N5	1.433 (4)
C7—N3	1.348 (3)	C16—H16A	0.9600
C7—N1	1.365 (3)	C16—H16B	0.9600
C8—C13	1.390 (4)	C16—H16C	0.9600
C8—C9	1.385 (4)	N1—N2	1.392 (3)
C8—N2	1.402 (3)	N1—H1A	0.8600
C9—C10	1.377 (4)	N2—H2B	0.8600
C9—H9A	0.9300	N3—N4	1.396 (3)
C10—C11	1.373 (4)	N3—H3B	0.8600
C10—H10A	0.9300	N4—H4B	0.8600
			
C2—C1—C6	118.6 (3)	C13—C12—H12A	119.5
C2—C1—N4	122.3 (2)	C12—C13—C8	120.1 (3)
C6—C1—N4	119.0 (2)	C12—C13—H13A	120.0
C3—C2—C1	120.2 (3)	C8—C13—H13A	120.0
C3—C2—H2A	119.9	O2—C14—N5	126.0 (3)
C1—C2—H2A	119.9	O2—C14—H14A	117.0
C4—C3—C2	121.0 (3)	N5—C14—H14A	117.0
C4—C3—H3A	119.5	N5—C15—H15A	109.5
C2—C3—H3A	119.5	N5—C15—H15B	109.5
C5—C4—C3	118.9 (3)	H15A—C15—H15B	109.5
C5—C4—H4A	120.6	N5—C15—H15C	109.5
C3—C4—H4A	120.6	H15A—C15—H15C	109.5
C6—C5—C4	120.9 (3)	H15B—C15—H15C	109.5
C6—C5—H5A	119.6	N5—C16—H16A	109.5
C4—C5—H5A	119.6	N5—C16—H16B	109.5
C5—C6—C1	120.5 (3)	H16A—C16—H16B	109.5
C5—C6—H6A	119.8	N5—C16—H16C	109.5
C1—C6—H6A	119.8	H16A—C16—H16C	109.5
O1—C7—N3	124.1 (2)	H16B—C16—H16C	109.5
O1—C7—N1	120.4 (2)	C7—N1—N2	119.9 (2)
N3—C7—N1	115.5 (2)	C7—N1—H1A	120.1
C13—C8—C9	118.8 (2)	N2—N1—H1A	120.1
C13—C8—N2	119.0 (2)	N1—N2—C8	118.7 (2)
C9—C8—N2	122.2 (2)	N1—N2—H2B	120.7
C10—C9—C8	120.0 (3)	C8—N2—H2B	120.7
C10—C9—H9A	120.0	C7—N3—N4	120.7 (2)
C8—C9—H9A	120.0	C7—N3—H3B	119.7
C11—C10—C9	121.0 (3)	N4—N3—H3B	119.7
C11—C10—H10A	119.5	N3—N4—C1	119.1 (2)
C9—C10—H10A	119.5	N3—N4—H4B	120.5
C10—C11—C12	119.1 (3)	C1—N4—H4B	120.5
C10—C11—H11A	120.5	C14—N5—C16	120.9 (2)
C12—C11—H11A	120.5	C14—N5—C15	120.9 (2)
C11—C12—C13	121.0 (3)	C16—N5—C15	118.0 (2)
C11—C12—H12A	119.5		
			
C6—C1—C2—C3	1.7 (4)	C9—C8—C13—C12	−0.9 (4)
N4—C1—C2—C3	177.9 (3)	N2—C8—C13—C12	−179.3 (2)
C1—C2—C3—C4	−0.7 (5)	O1—C7—N1—N2	170.8 (2)
C2—C3—C4—C5	−0.7 (5)	N3—C7—N1—N2	−8.7 (3)
C3—C4—C5—C6	1.0 (5)	C7—N1—N2—C8	−91.6 (3)
C4—C5—C6—C1	0.0 (4)	C13—C8—N2—N1	−173.7 (2)
C2—C1—C6—C5	−1.4 (4)	C9—C8—N2—N1	8.1 (3)
N4—C1—C6—C5	−177.7 (3)	O1—C7—N3—N4	11.1 (4)
C13—C8—C9—C10	0.7 (4)	N1—C7—N3—N4	−169.4 (2)
N2—C8—C9—C10	179.0 (2)	C7—N3—N4—C1	75.3 (3)
C8—C9—C10—C11	−0.3 (4)	C2—C1—N4—N3	19.4 (4)
C9—C10—C11—C12	0.0 (5)	C6—C1—N4—N3	−164.4 (2)
C10—C11—C12—C13	−0.2 (5)	O2—C14—N5—C16	−3.8 (5)
C11—C12—C13—C8	0.7 (4)	O2—C14—N5—C15	−179.1 (3)

### Hydrogen-bond geometry (Å, °)

**Table:**

*D*—H···*A*	*D*—H	H···*A*	*D*···*A*	*D*—H···*A*
N1—H1A···O1^i^	0.86	2.13	2.975 (3)	166
C6—H6A···O2^ii^	0.93	2.58	3.370 (4)	143
N2—H2B···O1^iii^	0.86	2.45	3.121 (3)	135
N3—H3B···O2	0.86	2.12	2.895 (3)	149
N4—H4B···O2^ii^	0.86	2.31	3.079 (3)	148

Symmetry codes: (i) −*x*+1, −*y*+1, −*z*; (ii) *x*−1, *y*, *z*; (iii) *x*+1, *y*, *z*.
